# Nasal Methicillin-Resistant *Staphylococcus aureus* Colonization in Patients with Type 1 Diabetes in Taiwan

**DOI:** 10.3390/microorganisms9061296

**Published:** 2021-06-15

**Authors:** Chun-Ya Kang, Eugene Yu-Chuan Kang, Chi-Chun Lai, Wei-Che Lo, Kun-Jen Chen, Wei-Chi Wu, Laura Liu, Yih-Shiou Hwang, Fu-Sung Lo, Yhu-Chering Huang

**Affiliations:** 1 School of Medicine, Medical University of Lublin, 20529 Lublin, Poland; miranda52879@gmail.com; 2 College of Medicine, Chang Gung University, Taoyuan 333, Taiwan; yckang0321@gmail.com (E.Y.-C.K.); chichun.lai@gmail.com (C.-C.L.); cgr999@gmail.com (K.-J.C.); weichi666@gmail.com (W.-C.W.); laurajl@gmail.com (L.L.); yihshiou.hwang@gmail.com (Y.-S.H.); 3 Department of Ophthalmology, Chang Gung Memorial Hospital, Linkou Medical Center, Taoyuan 333, Taiwan; 4 Department of Family Medicine, National Taiwan University Hospital, Taipei 100, Taiwan; 5 Department of Ophthalmology, Keelung Chang Gung Memorial Hospital, Keelung 204, Taiwan; b01401103@ntu.edu.tw; 6 Division of Pediatric Endocrinology and Genetics, Chang Gung Memorial Hospital, Linkou Medical Center, Taoyuan 333, Taiwan; 7 Division of Pediatric Infectious Diseases, Department of Pediatrics, Chang Gung Memorial Hospital, Linkou Medical Center, Taoyuan 333, Taiwan

**Keywords:** *Staphylococcus aureus*, methicillin-resistant, nasal colonization, type 1 diabetes, molecular, epidemiology

## Abstract

Nasal methicillin-resistant *Staphylococcus aureus* (MRSA) colonies are an essential reservoir of infection, especially for patients with diabetes. However, data on MRSA colonization in patients with type 1 diabetes are limited. We investigated the epidemiology of MRSA colonization in patients with type 1 diabetes. This prospective cross-sectional study was conducted in a medical center (Chang Gung Memorial Hospital) in Taiwan from 1 July to 31 December 2020. Nasal sampling and MRSA detection were performed. The molecular characteristics of MRSA isolates were tested, and factors associated with MRSA colonization were analyzed. We included 245 patients with type 1 diabetes; nasal MRSA colonization was identified in 13 (5.3%) patients. All isolates belonged to community-associated MRSA genetic strains; the most frequent strain was clonal complex 45 (53.8%), followed by ST59 (30.8%) (a local community strain). MRSA colonization was positively associated with age ≤ 10 years, body mass index < 18 kg/m^2^, and diabetes duration < 10 years; moreover, it was negatively associated with serum low-density lipoprotein cholesterol ≥ 100 mg/dL. No independent factor was reported. The nasal MRSA colonization rate in type 1 diabetes is approximately 5% in Taiwan. Most of these colonizing strains are community strains, namely clonal complex 45 and ST59.

## 1. Introduction

*Staphylococcus aureus* is an essential cutaneous pathogen of serious infections in humans and causes a wide range of diseases [1,2]. It is vital to understand the pathogenesis of *S. aureus* infections and develop new approaches to prevent the disease. Via its adhesive and invasive ability, *S. aureus* can harbor in human tissue and create biofilms [3,4], as well as secret several virulence factors that can impair human immunity [5]. Colonizing strains are endogenous reservoirs for *S. aureus* infections, with anterior nares being one of the principal carriage sites [6], and the host’s characteristics could influence the colonization [3].

The nasal carriage rate of *S. aureus* is approximately 30% in humans, and the elimination of carriage in these people decreases the rate of subsequent *S. aureus* infections [2,7]. Methicillin-resistant *S. aureus* (MRSA) was first described in 1961 and is increasingly discovered worldwide [8]. MRSA is now prevalent in most hospitals and accounts for 50%–80% of nosocomial *S. aureus* infections [9]. It is challenging to treat because it is resistant to numerous antibiotics. MRSA is a clinical threat not only because of its high prevalence and difficulty in management but also because of its high morbidity and mortality [10]. Given its poor prognosis, identifying and managing MRSA carriage are critical to reducing the chances of unfavorable outcomes. In Taiwan, MRSA was first found in the early 1980s and rapidly spread in the 1990s [11]. Community-associated MRSA (CA-MRSA) infections, as well as the nasal MRSA carriage rate, have continued to rise [12,13,14]. With the increase in the nasal MRSA carriage rate, an increase in the rate of MRSA in children was also reported [13].

Type 1 diabetes is a major health issue in the pediatric population, and the number of children with this disease has been increasing in recent years [15]. Type 1 diabetes leads to several systemic complications not only in the vascular system but also in the immune system [16], thus increasing the risk of infection [17]. Patients with type 1 diabetes also have altered cutaneous conditions [18], which may increase their susceptibility to cutaneous *S. aureus* or MRSA infection. Additionally, the autoimmune process involved in type 1 diabetes may alter the patients’ innate immune system [19], which plays an important role in the defense mechanism against *S. aureus* colonization or infection [20]. Several other factors, such as angiopathy and neuropathy, in diabetic patients were reported to predispose them to infection, and staphylococcal infection could be found in diabetic foot ulcers and necrotizing fasciitis [21]. Patients with type 1 diabetes are often exposed to healthcare facilities for follow-up visits and prescription refills, and they frequently receive subcutaneous insulin injections [22,23], though the correlation of the subcutaneous insulin injection and MRSA colonization or infection remains unclear. Based on the aforementioned property in patients with type 1 diabetes, skin MRSA colonization is an important issue in this population.

However, few studies have analyzed the rate of MRSA carriage in patients with type 1 diabetes mellitus. Therefore, we investigated the prevalence of nasal MRSA colonization among patients with type 1 diabetes to delineate molecular characteristics and antimicrobial resistance profiles of MRSA and analyze the demographic and clinical characteristics associated with MRSA colonization in Taiwan.

## 2. Materials and Methods

### 2.1. Study Population and Clinical Data Collection

This prospective study was conducted from 15 July to 31 December 2020, at Chang Gung Memorial Hospital, Linkou Medical Center, a 3700-bed referral hospital and one of the largest medical centers in Taiwan. We invited patients with confirmed type 1 diabetes receiving insulin supplements to participate during clinical follow-up at the Department of Pediatrics or the Department of Ophthalmology. All the patients were from the Chang Gung Juvenile Diabetes Eye Study, which has been published previously [24,25], and diagnosed as type 1 diabetes according to the clinical criteria recommended by the World Health Organization [26]. Patient characteristics, including age, sex, height, weight, body mass index, date of diabetes diagnosis, and underlying diseases, were recorded. Blood tests, including hemoglobin A1c (HbA1c), creatinine, high-density lipoprotein cholesterol (HDL-C), low-density lipoprotein cholesterol (LDL-C), total cholesterol, and triglyceride, were performed. All patients or their legal guardians were requested to complete written consent before participating in the study. The study was approved by the Chang Gung Memorial Hospital Institutional Review Board (No. 202001183B0), and the study adhered to the principles of the Declaration of Helsinki.

### 2.2. Sampling

For each patient, a nasal specimen was obtained from the anterior nares using a cotton swab, and the swab was placed immediately into the transport medium (Venturi Transystem; Copan Innovation, Copan Diagnostics, Murrieta, CA, USA). The collected samples were then inoculated overnight using streak plate methods onto Trypticase soy agar plates containing 5% sheep blood (Becton, Dickinson and Company, Sparks, MD, USA). The strains grown on the agar plates were evaluated for morphology, Gram stain, and coagulase tests, and *S. aureus* was identified based on the results.

### 2.3. Antibiotic Susceptibility

All *S. aureus* isolates were tested for antibiotic susceptibility to cefoxitin, clindamycin, erythromycin, fusidic acid, penicillin, doxycycline, sulfamethoxazole–trimethoprim, linezolid, teicoplanin, and vancomycin using the disk-diffusion method based on the guidelines of Clinical and Laboratory Standard Institutes [27]. MRSA was defined as *S. aureus* with β-lactam antibiotic resistance according to the cefoxitin susceptibility result. E-test (BioMerieux SA, Marcy-I’Etoile, France) was used for testing antibiotic susceptibility to ciprofloxacin.

### 2.4. Molecular Typing

We analyzed all MRSA strains for their molecular characteristics, including pulsed-field gel electrophoresis (PFGE) pulsotype, multilocus sequence type (MLST), staphylococcal cassette chromosome *mec* (SCC*mec*) typing, *Spa* gene typing, and the presence of Panton–Valentine leukocidin (PVL) genes. PFGE was performed with Smal digestion methods, and the pulsotypes were designated as in our previous studies [12,28]. If the PFGE patterns had fewer than four band differences compared with an existing pulsotype, the strain was defined as a subtype of the pulsotype. MLST, SCC*mec*, and *Spa* gene typing were performed as described previously [29,30,31]. PVL genes were identified using polymerase chain reaction [32,33].

### 2.5. Statistical Analysis

Categorical variables are indicated as numbers and percentages, and continuous variables are indicated as mean ± standard deviation. For descriptive statistics, the chi-square test, Fisher’s exact test, and Student’s *t*-test were used as appropriate. Risk analysis was performed using binary regression for univariate and multivariate analysis, and the odds ratios (ORs) and 95% confidence intervals (95% CIs) were calculated. Results with *p* < 0.05 were considered statistically significant. All statistical analyses were performed using IBM SPSS Statistics for Windows 19.0 (IBM Corp., Armonk, NY, USA).

## 3. Results

### 3.1. Colonization and Patient Characteristics

Overall, 245 patients with type 1 diabetes (age range: 4–45 years) were included. Of them, 129 (52.7%) were female. The duration of diabetes was calculated by the date of diabetes diagnosis and sampling, and the average duration of diabetes was 14.4 ± 6.2 years. Among the 245 patients, 78 (31.8%) had *S. aureus* colonization, and 13 (5.3%) of them had MRSA isolates. The patients with MRSA colonization were younger (14.4 years vs. 22.9 years, *p* < 0.001) and had shorter diabetes duration (8.4 years vs. 14.8 years, *p* < 0.001), lower body weight (42.5 kg vs. 59.6 kg, *p* < 0.001), shorter height (141 cm vs. 161.5 cm, *p* = 0.002), lower body mass index (19.6 kg/m^2^ vs. 22.9 kg/m^2^, *p* = 0.037), and lower serum creatinine level (0.45 mg/dL vs. 0.64 mg/dL, *p* < 0.001). MRSA colonization was especially predominant in patients aged ≤10 years (46.2% vs. 5.6%, *p* = 0.001). No difference was observed in sex, hypertension, or HbA1c, HDL-C, LDL-C, total cholesterol, and triglyceride levels. The demographic characteristics of patients with and without MRSA nasal colonization are presented in Table 1.

### 3.2. Antibiotic Susceptibility

The antibiotic profiles of the MRSA isolates are demonstrated in Table 2. All strains were susceptible to fusidic acid, linezolid, sulfamethoxazole–trimethoprim, teicoplanin, vancomycin, and doxycycline, and all were resistant to penicillin. The susceptibility rates of the MRSA isolates to both clindamycin and ciprofloxacin were 92.3% (12/13), and the susceptibility to erythromycin was 54.0% (7/13).

### 3.3. Molecular Characteristics

**Figure 1:**
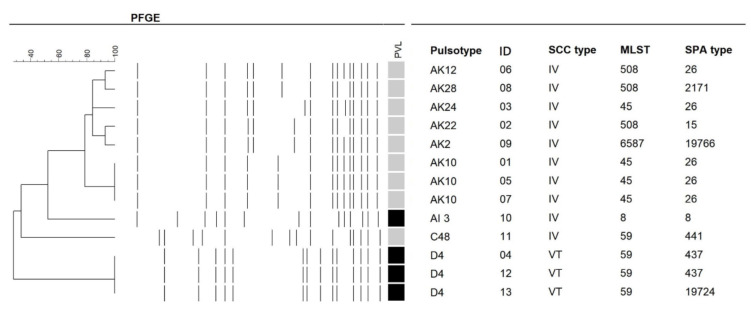
Molecular characteristics of 13 methicillin-resistant *Staphylococcus aureus* isolates. Black color indicates positive. ST508 is a single-locus variant of ST45, and ST6587 is a single-locus variant of ST508. PFGE: pulsed-field gel electrophoresis; SCC*mec*: staphylococcal cassette chromosome *mec*; PVL: Panton–Valentine leukocidin; MLST, multilocus sequence type; *Spa*: staphylococcal protein A.

Figure 1 presents the PVL genes, PFGE patterns, MLSTs, SCC*mec* types, and *Spa* gene typing in the 13 MRSA strains. Four (30.8%) isolates were PVL positive. For PGFE patterns, pulsotype AK had the highest frequency (61.5%), followed by pulsotype D (23.1%), C (7.7%), and AI (7.7%). Only two types (types IV and VT) of SCC*mec* were identified in our study, of which type IV was predominant (76.9%). Five clonal lineages were identified by MLST, of which ST45 (30.8%) and ST59 (30.8%) were predominant, followed by ST508 (23.1%) and ST8 (7.7%). ST508 is a single-locus variant of ST45 and was classified as clonal complex 45 (CC45) [34]; 7 of 13 (53.8%) strains belonged to CC45 in our study. *Spa* gene typing 26 had the highest frequency (38.5%) and was found in the pulsotype AK/ST45 or ST508/SCC*mec* IV/PVL negative. One novel strain with pulsotype AK/ST 6587/SCC*mec* IV/*Spa* type 19766/PVL negative was identified in this study, which is a single-locus variation of ST508 (in the yqiL allele).

### 3.4. Factor Analysis

Table 3 summarizes the factors associated with MRSA colonization. Univariate analysis revealed that MRSA colonization was positively associated with age ≤ 10 years (OR: 14.44, 95% CI: 4.24–49.18), body mass index < 18 kg/m^2^ (OR: 7.10, 95% CI: 2.21–22.79), and diabetes duration < 10 years (OR: 8.86, 95% CI: 2.61–30.01) and negatively associated with age > 10 years (OR: 0.07, 95% CI: 0.02–0.44), diabetes duration ≥ 10 years (OR: 0.12, 95% CI: 0.03–0.39), and elevated LDL-C (OR: 0.27, 95% CI: 0.08–0.89), which was defined as serum LDL-C ≥ 100 mg/dL. However, in multivariate analysis, no independent factor was found to be associated with MRSA colonization (all *p* > 0.05).

## 4. Discussion

In the present study, we investigated the epidemiology of nasal MRSA carriage in 245 patients with type 1 diabetes. The nasal MRSA colonization rate was 5.3% in our cohort, which was mainly CA-MRSA strains. Strains belonging to CC45 had the highest frequency. Younger age, shorter diabetes duration, and lower body mass index were positively associated with nasal MRSA colonization. We also found one novel strain with pulsotype AK/ST 6587/SCC*mec* IV/*Spa* type19766/PVL negative.

According to studies from Taiwan, nasal MRSA carriage is observed in approximately 3.8% of health examinations for the general population [35] and adult patients visiting emergency departments [36]. In adults with type 2 diabetes, the nasal MRSA colonization rate was reported to be 2.8% overall and approximately 5.4% in the subgroup with diabetic foot ulcers [37]. In the pediatric population, the colonization rates ranged from 10.2% in infants [38] to 7.8% in healthy school-age children visiting hospitals in Taiwan [39]. According to a meta-analysis using global data, the MRSA colonization rate in children with any underlying condition is 5.4% [40]. Although the epidemiology of nasal MRSA colonization in children has been widely studied in different populations [40,41], the investigation of nasal MRSA colonization in patients with type 1 diabetes has been limited. In Turkey, Karadag-Oncel et al. reported nasal MRSA colonization rates of 0.7% in 2005 and 0.9% in 2013 [42]. No studies have evaluated this in Asian countries.

To the best of our knowledge, this is the first study exploring nasal MRSA colonization in patients with type 1 diabetes in Asia. The colonization rate of 5.3% was in between those identified in the general adult and pediatric populations in Taiwan and is comparable with that of children with underlying conditions (5.4%) [40]. However, the rate is much higher than that reported in patients with type 1 diabetes in Turkey (0.7% to 0.9%) [42]. The difference might be related to the relatively low nasal MRSA colonization rate in healthy adults (0.37%) and children (0.07%) in Turkey [43,44]. The relatively higher MRSA colonization rate in patients with type 1 diabetes compared to healthy children may contribute to the host environment. Previous investigations have indicated the pathophysiology of type 1 diabetes involving the immune system, especially innate immunity [19,45], which could affect the *S. aureus* and MRSA colonization and subsequent infection [20,46].

In our study, all isolates belonged to CA-MRSA genetic strains and shared similar antibiotic susceptibility patterns [13]. The results suggested that the characteristics of patients with type 1 diabetes were similar to those of the community population in the aspect of MRSA colonization, though the patients with type 1 diabetes may be exposed to healthcare facilities more frequently. The clonal spread of molecular CA strains to healthcare-associated (HA) environments has been noticed in recent years [28,47]. Although the population may embrace HA factors, increased colonization of the molecular CA strains may indicate a changing MRSA epidemiology in the community and healthcare settings [48].

The 13 MRSA isolates all carried either SCC*mec* type IV or VT, indicating CA-MRSA strains [13,49]. For MLST typing, ST59 (30.8%) and ST45 (30.8%) were the most frequent strains, followed by ST508 (23.1%). ST59 was the most common (>80%) endemic CA-MRSA strain in Taiwan, which was reported to be especially high in children [13]. Two distinct types were classified: a virulent Taiwan clone (pulsotype D/ST59/SCC*mec* VT/PVL positive) and a commensal Asia-Pacific clone (pulsotype C/ST59/SCC*mec* IV/PVL negative) [49,50,51], both of which were identified in our study. Furthermore, we found a high frequency of strains with pulsotype AK/ST45/SCC*mec* IV/PVL negative, which were first identified in 2006 in Taiwan and reportedly predominant in immigrant workers from southeast Asian countries [52,53]. In recent years, this strain has been increasing in Taiwanese children [51]. ST508, which is a single-locus variation of ST45 and was classified as CC45, was previously reported more commonly in methicillin-susceptible *S. aureus* [54]. Nevertheless, the emergence of ST508 in MRSA has been noted in Taiwan in recent years [34], and ST45 was also increasingly reported in MRSA isolates [55]. In the present study, one novel strain was discovered (pulsotype AK/ST 6587/SCC*mec* IV/*Spa* type 19766/PVL negative). This strain had a single-locus variation compared with ST508 and may be considered a CC45 variant. It was resistant to erythromycin and penicillin but susceptible to the other antibiotics tested; the pattern was similar to the CA-MRSA strains in Taiwan [13]. The clinical significance and influence of this strain may require further research.

On the basis of the risk analysis, younger age, lower body mass index (<18 kg/m^2^), and diabetes duration < 10 years were found to be positively associated with MRSA colonization. Additionally, the colonization rate was especially high in patients aged ≤10 years. However, multivariate analysis revealed that no independent factor was associated with MRSA colonization, indicating that the factors may be correlated with each other. For example, younger children have a lower body mass and shorter diabetes duration. Younger age has been reported as a risk factor for MRSA colonization in Chinese children [41], and MRSA colonization rates are higher in the younger population in Taiwan comparing to those in the overall population [38,39]. However, this phenomenon has not been reported in other countries [56,57]. This controversial finding may need further investigation in a population with a wider age range. Although a change in lipid profile was correlated to age in the pediatric population [58], only the serum LDL-C level was negatively associated with MRSA colonization in our study. Serum LDL-C serves as a nutrient source for *S. aureus* in the human body [59]; by contrast, other studies have reported that LDL-C might bind and inactivate the protein function in *S. aureus* [60,61,62], thus attenuating its infectiousness. However, it is uncertain whether LDL-C may prevent *S. aureus* colonization on the skin.

Antimicrobial susceptibility was similar to that of a previous report regarding CA-MRSA strains in Taiwan [13]. However, the susceptibility rates of clindamycin (92.3%) and erythromycin (54.0%) were higher than those reported previously (0%–51% for clindamycin and 0%–22% for erythromycin) [13,38,63]. On the basis of our previous observation, the susceptibility to clindamycin and erythromycin has been increasing since 2005 [28,51], which may indicate a changing characteristic of CA-MRSA clones in Taiwan.

This study has some limitations. First, we only obtained one specimen from the patient’s nostril at one time point, so the carriage rate may have been underestimated in this population. Second, we did not observe the longitudinal change in colonization or any subsequent infectious events. Third, the patient population included both pediatric and adult patients, so confounding effects due to age differences may have occurred in risk analysis. Fourth, the single-center design precludes the generalizability of the findings to other hospitals and countries. Finally, because the number of patients in this study was relatively low, the statistical tests may be underpowered. A large-scale multi-center prospective study is warranted for further and more definitive risk identification.

## 5. Conclusions

In conclusion, this is the first study demonstrating an MRSA nasal colonization rate of 5.3% in patients with type 1 diabetes in Taiwan. Molecular analysis revealed that CA-MRSA strains with CC45 were predominant in this population. Younger age, shorter diabetes duration, and lower body mass index were positively associated with MRSA colonization.

## Figures and Tables

**Table 1:** Demographics of patients with type 1 diabetes.

Variates	Non-MRSA (n = 232)	MRSA (n = 13)	Total (n = 245)	*p* Value *
Male (no.) (%)	112 (48.3)	4 (30.8)	116 (47.3)	0.219
Age (year) (mean ± SD)	22.9 ± 6.5	14.4 ± 8.4	22.4 ± 6.8	<0.001
Age ≤ 10 years (%)	13 (5.6)	6 (46.2)	19 (7.8)	0.001
Age > 10 years (%)	219 (94.4)	7 (53.8)	226 (92.2)	
Diabetes duration (year) (mean ± SD)	14.8 ± 6.0	8.4 ± 6.6	14.4 ± 6.2	<0.001
HbA1c (%) (mean ± SD)	8.6 ± 2.0	8.4 ± 1.3	8.6 ± 2.0	0.714
Body weight (kg) (mean ± SD)	59.6 ± 14.1	42.5 ± 16.0	58.8 ± 14.6	<0.001
Height (cm) (mean ± SD)	161.5 ± 12.7	141.7 ± 18.6	160.4 ± 13.7	0.002
Body mass index (kg/m^2^) (mean ± SD)	22.9 ± 0.3	19.6 ± 1.1	22.8 ± 5.6	0.037
Hypertension (no.) (%)	38 (16.4)	1 (7.7)	39 (15.9)	0.699
Serum creatinine (mg/dL) (mean ± SD)	0.64 ± 0.16	0.45 ± 0.12	0.63 ± 0.16	<0.001
Serum HDL-C (mg/dL) (mean ± SD)	62.6 ± 15.5	70.5 ± 16.6	63.0 ± 15.6	0.073
Serum LDL-C (mg/dL) (mean ± SD)	111.0 ± 34.1	95.5 ± 29.6	110.2 ± 34.0	0.110
Serum total cholesterol (mg/dL) (mean ± SD)	187.0 ± 42.0	181.2 ± 33.8	186.7 ± 41.6	0.625
Serum triglyceride (mg/dL) (mean ± SD)	81.4 ± 61.4	63.5 ± 41.3	80.5 ± 60.6	0.301

MRSA: methicillin-resistant *Staphylococcus aureus*; SD: standard deviation; HbA1c: hemoglobin A1c; HDL-C: high-density lipoprotein cholesterol; LDL-C: low-density lipoprotein cholesterol. * *p* value was obtained by chi-square test or Fisher’s exact test (when any expected count was less than 5) for categorial variates and by Student’s *t*-test for continuous variates.

**Table 2:** Antibiotic susceptibility of nasal methicillin-resistant *Staphylococcus aureus* colonies.

ID	CC	E	FA	LZD	P	SXT	TEC	VA	CIP	D
01	S	S	S	S	R	S	S	S	S	S
02	S	S	S	S	R	S	S	S	S	S
03	S	R	S	S	R	S	S	S	S	S
04	S	S	S	S	R	S	S	S	S	S
05	S	R	S	S	R	S	S	S	S	S
06	S	S	S	S	R	S	S	S	S	S
07	S	S	S	S	R	S	S	S	S	S
08	S	R	S	S	R	S	S	S	S	S
09	S	R	S	S	R	S	S	S	S	S
10	S	R	S	S	R	S	S	S	R	S
11	R	R	S	S	R	S	S	S	S	S
12	S	S	S	S	R	S	S	S	S	S
13	S	S	S	S	R	S	S	S	S	S

CC: clindamycin; E: erythromycin; FA: fusidic acid; LZD: linezolid; P: penicillin; SXT: sulfamethoxazole–trimethoprim; TEC: teicoplanin; VA: vancomycin; CIP: ciprofloxacin; D: doxycycline; S: susceptible; R: resistant.

**Table 3:** Univariate and multivariate analysis of clinical characteristics associated with methicillin-resistant *Staphylococcus aureus* colonization.

	Univariate Analysis			Multivariate Analysis		
	Odds Ratio	95% CI	*p* Value	Odds Ratio	95% CI	*p* Value
Male	0.48	0.14–1.59	0.23			
Age						
≤10 years	14.44	4.24–49.18	<0.001	7.40	0.69–79.47	0.099
>10 years	0.07	0.02–0.24	<0.001			
Body mass index						
<18 kg/m^2^	7.10	2.21–22.79	0.001	0.63	0.06–6.36	0.698
19–24 kg/m^2^	0.43	0.14–1.37	0.153			
>24 kg/m^2^	0.44	0.10–2.03	0.292			
Diabetes duration						
<10 years	8.86	2.61–30.01	<0.001	3.92	0.81–18.87	0.089
≥10 years	0.12	0.03–0.39	0.001			
HbA1c						
<7.0%	0.74	0.16–3.43	0.696			
7.0%–10.0%	2.11	0.57–7.89	0.266			
>10%	0.36	0.05–2.81	0.327			
Hypertension	0.43	0.05–3.37	0.418			
Elevated LDL-C ^1^	0.27	0.08–0.89	0.032	0.29	0.08–1.10	0.068
Elevated total cholesterol ^2^	0.93	0.30–2.94	0.907			
Elevated triglyceride ^3^	0.84	0.10–6.76	0.868			

CI: confidence interval; HbA1c: hemoglobin A1c; LDL-C: low-density lipoprotein cholesterol. ^1^ Defined as serum LDL-C level ≥ 100 mg/dL. ^2^ Defined as serum total cholesterol level ≥ 170 mg/dL in patients aged younger than 18 years and ≥ 200 mg/dL in patients aged older than 18 years. ^3^ Defined as serum triglyceride level ≥ 110 mg/dL in patients aged younger than 9 years and ≥ 150 mg/dL in patients aged older than 9 years.

## Data Availability

The data presented in this study are available on request. The data are not publicly available due to the data security policy of Chang Gung Memorial Hospital.
